# The Clinical Relevance of the miR-197/CKS1B/STAT3-mediated PD-L1 Network in Chemoresistant Non-small-cell Lung Cancer

**DOI:** 10.1038/mt.2015.10

**Published:** 2015-02-10

**Authors:** Yu Fujita, Shigehiro Yagishita, Keitaro Hagiwara, Yusuke Yoshioka, Nobuyoshi Kosaka, Fumitaka Takeshita, Tomohiro Fujiwara, Koji Tsuta, Hiroshi Nokihara, Tomohide Tamura, Hisao Asamura, Makoto Kawaishi, Kazuyoshi Kuwano, Takahiro Ochiya

**Affiliations:** 1 Division of Molecular and Cellular Medicine, National Cancer Center Research Institute, Tokyo, Japan; 2 Division of Respiratory Diseases, Department of Internal Medicine, Jikei University School of Medicine, Tokyo, Japan; 3 Department of Thoracic Oncology, National Cancer Center Hospital, Tokyo, Japan; 4 Department of Pathology, National Cancer Center Hospital, Tokyo, Japan; 5 Department of Thoracic Surgery, National Cancer Center Hospital, Tokyo, Japan

## Abstract

Programmed cell death ligand-1 (PD-L1) has recently gained considerable attention for its role in tumor immune escape. Here, we identify a miR-197/CKS1B/STAT3-mediated PD-L1 network in chemoresistant non-small-cell lung cancer (NSCLC), independent of immunoinhibitory signals. miR-197 is downregulated in platinum-resistant NSCLC specimens, resulting in the promotion of chemoresistance, tumorigenicity, and pulmonary metastasis *in vitro* and *in vivo*. Mechanistic investigations reveal that a miR-197-mediated CKS1B/STAT3 axis exerts tumor progression regulated by various oncogenic genes (Bcl-2, c-Myc, and cyclin D1), and PD-L1 is a putative biomarker of this axis. Furthermore, we demonstrate that a miR-197 mimic sensitizes PD-L1^high^ drug-resistant cells to chemotherapy. These results indicate that the biological interaction between PD-L1 and chemoresistance occurs through the microRNA regulatory cascade. More importantly, expression levels of miR-197 are inversely correlated with PD-L1 expression (*n* = 177; *P* = 0.026) and are associated with worse overall survival (*P* = 0.015). Our discoveries suggest that the miR-197/CKS1B/STAT3-mediated network can drive tumor PD-L1 expression as a biomarker of this cascade, and miR-197 replacement therapy may be a potential treatment strategy for chemoresistant NSCLC.

## Introduction

Lung cancer is the leading cause of cancer-related deaths. Approximately 70% of all newly diagnosed patients present with local advanced or metastatic disease and require systemic chemotherapy.^1^ Patients with non-small-cell lung cancer (NSCLC) are typically treated with platinum-based chemotherapy. Despite the development of novel targeted therapies, the prognosis of lung cancer remains poor due to drug resistance and tumor recurrence. Understanding the critical molecular mechanisms underlying the development of chemoresistance in NSCLC is an important issue for developing novel and effective therapeutic strategies.

MicroRNAs (miRNAs), a family of short endogenous noncoding RNAs, play critical roles in cell growth, differentiation, and the development of various solid and hematological malignancies.^2^ Recently, miRNAs have emerged in NSCLC as both diagnostic and prognostic biomarkers.^3^ These findings suggest that miRNAs are a promising technology for the development of therapeutic targets for lung cancer. However, there is little evidence of miRNA-mediated signaling networks that regulate cancer progression and drug resistance in NSCLC.

Currently, the launch of an antiprogrammed cell death ligand-1 (PD-L1) antibody represented a significant breakthrough for patients with advanced solid tumors.^4^ Strikingly, blockade of PD-L1 induced durable tumor regression and prolonged disease stabilization in patients with advanced NSCLC. This successful immunotherapeutic strategy is a next-generation approach to the treatment of PD-L1-positive NSCLC. Although many studies have focused on the relationship between PD-L1 and immune escape, there are limited data on constitutive upstream signaling pathways of PD-L1, independent of immunoinhibitory activities in the NSCLC microenvironment.^5^

Here, we show the biological and clinical function of PD-L1 expression in chemoresistant NSCLC through the miRNA regulatory cascade. We identify a miR-197, a NSCLC prognostic marker, has a pivotal role in platinum-based chemotherapy resistance by analyzing clinical specimens of NSCLC. Further investigations show that PD-L1 can serve as a putative biomarker of the miR-197/CKS1B/STAT3-mediated pathway in NSCLC. Moreover, we demonstrate the clinical significance of the miR-197/PD-L1 network in tumor specimens from NSCLC patients. Our discoveries provide novel insights regarding the therapeutic potential of miR-197 and introduce novel mechanisms regulating cancer progression in PD-L1-positive NSCLC.

## Results

### Downregulation of miR-197 in NSCLC is associated with chemoresistance and survival

**Figure 1:**
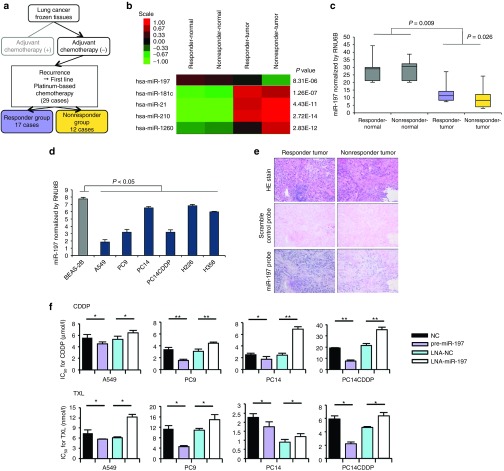
**miR-197 is a novel miRNA related to chemoresistance and survival in NSCLC.** (**a**) Schema for the patient selection of a cohort study for miRNA microarray. (**b**) A heat map of miRNA microarray analysis revealed differentially expressed miRNAs in tumor and adjacent normal tissues in the two groups. (**c**) qRT-PCR analysis of the expression levels of miR-197 in lung tumors and adjacent normal tissues. (**d**) qRT-PCR analysis of the expression levels of miR-197 in human lung cancer cell lines. (**e**) miR-197-specific probe and scramble control probe were hybridized *in situ* with lung adenocarcinoma tissue. Original magnification, ×100. (**f**) IC_50_ after transient transfection of each miRNA and CDDP and TXL treatment. *P < 0.05; **P < 0.01. CDDP, cisplatin; HE, hematoxylin and eosin; IC_50_, concentration of drug needed to inhibit cell growth by 50%; LNA, locked nucleic acid; miRNA, microRNA; NC, negative control; NSCLC, non-small-cell lung cancer; qRT-PCR, quantitative reverse transcription-PCR; TXL, paclitaxel.

To identify miRNAs that regulate chemoresistance, we conducted a high-throughput miRNA microarray on a cohort study of primary NSCLC tissues and adjacent normal tissues (**Figure 1a**). Twenty-nine patients who received four or more courses of first-line platinum-based chemotherapy after postoperative recurrence were divided into two groups (responder (*n* = 17) and nonresponder group (*n* = 12)) (**Supplementary Table S1**). After analysis of variance of miRNA microarray signal expression from the tumor and adjacent normal tissues of the two groups, 17 miRNAs (6 upregulated miRNAs and 11 downregulated miRNAs in nonresponder's tumors) exhibited significant expression changes (**Supplementary Figure S1a**). Next, quantitative reverse transcription-PCR (qRT-PCR) validation of tumor samples from the two groups (**Supplementary Figure S1a**) showed that five miRNAs (miR-197, miR-181c, miR-21, miR-210, and miR-1260) exhibited significant expression changes (**Figure 1b** and **Supplementary Figure S1b**). To determine the role of the expression of these miRNAs in lung cancer prognosis, we first examined their clinical relevance to patient survival. Consequently, we identified miR-197 and miR-1260 expression as favorable lung cancer prognostic markers for overall survival (**Supplementary Figure S1c**). From the differentially regulated putative miRNA prognostic biomarkers, we focused on the tumor suppressor miRNA—miR-197. We validated this finding using qRT-PCR for miR-197 in the lung tumors and adjacent normal tissues of the cohort study (**Figure 1c**). An analysis of miR-197 expression levels in a panel of human lung cancer cell lines revealed that miR-197 was significantly suppressed in lung cancer cell lines compared with a normal bronchial cell line, BEAS-2B. Moreover, we observed that the expression of miR-197 in drug-resistant cell line PC14/cisplatin (CDDP) was lower than that in the parental lung cancer cell line PC14 (**Figure 1d**). In agreement with the qRT-PCR analysis, *in situ* hybridization with a miR-197-locked nucleic acid (LNA) probe revealed reduced miR-197 expression in the sample of nonresponders compared with responder samples (**Figure 1e**). We hypothesized that miR-197 might functionally regulate the therapeutic response in addition to its association with treatment benefits. Therefore, we focused on the role of miR-197 in regulating chemoresistance in NSCLC. To determine whether miR-197 plays a role in lung cancer progression and chemoresistance, we transiently transfected LNA-miR-197 or pre-miR-197 into four independent lung cancer cell lines-A549, PC9, PC14, and PC14CDDP. Interestingly, the knockdown of miR-197 induced significant resistance to CDDP and paclitaxel (TXL) in the four independent lung cancer cell lines. Furthermore, the overexpression of miR-197 sensitized the drug response of these compounds *in vitro* (**Figure 1f**).

### Stable knockdown of miR-197 promotes malignancy *in vitro*

**Figure 2:**
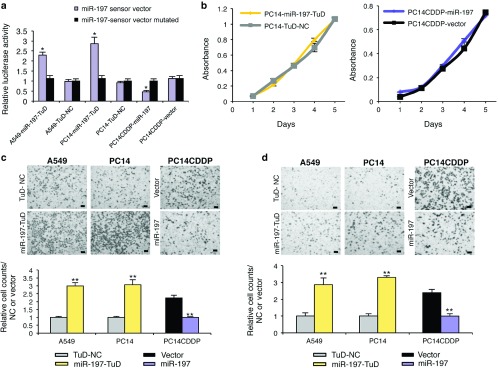
**miR-197 regulates malignant phenotypes of lung cancer cells *in vitro*.** (**a**) The luciferase activity of A549-miR-197-TuD, PC14-miR-197-TuD, and PC14CDDP-miR-197 cells was compared to that of each control cell line in response to a miR-197 sensor vector or the mutated vector. (**b**) No significant effect of decreased or increased miR-197 levels on the growth rate of PC14 or PC14CDDP. (**c** and **d**) Representative images (upper panels, pictures) and quantification (lower panels, graphs) of the effect on (**c**) cell invasion and (**d**) migration. The invasive or migration values were normalized to the values from control cells (scale bar = 100 μm). *P < 0.05; **P < 0.01. NC, negative control.

Having demonstrated the suppression of miR-197 expression in NSCLC, we sought to evaluate the other roles of the miRNA *in vitro*. We constructed a Tough Decoy (TuD) vector expressing a miR-197-TuD strategy for A549 and PC14 cells, which has previously been used for miRNA blockade,^6^ to stably suppress miR-197 expression. We also established PC14CDDP cells with cytomegalovirus promoter-mediated overexpression of miR-197 (PC14CDDP-miR-197). In miR-197 sensor assays, to confirm stable miRNA expression, we observed that the luciferase activity of A549-miR-197-TuD and PC14-miR-197-TuD cells was significantly increased compared with each negative control cell line and mutated sensor vector, indicating that miR-197 expression in these cell lines was functionally repressed. The activity of PC14CDDP-miR-197 cells was significantly reduced, indicating that miR-197 expression of the cells was functionally activated (**Figure 2a**). Next, we evaluated the effects of miR-197 on cell growth, cell invasion, cell migration, and spheroid initiation. Cell proliferation analysis suggested that decreased or increased miR-197 levels did not affect cancer cell growth (**Figure 2b**). Conversely, miR-197 loss of function enhanced the invasion and migration rates of A549 and PC14 cells. PC14CDDP cells stably overexpressing miR-197 exhibited suppressed invasion and migration rates compared with that of controls (**Figure 2c**,**d**). In addition, we evaluated A549-miR-197-TuD, PC14-miR-197-TuD, and PC14CDDP-miR-197 cells for their ability to form tumor spheres. A549-miR-197-TuD and PC14-miR-197-TuD generated two to threefold more spheres than the corresponding control cells, and the spheroid initiation of PC14CDDP-miR-197 cells was repressed compared with that of control cells (**Supplementary Figure S2a**). PC14-miR-197-TuD cells expressed high levels of various lung cancer initiation-related genes, particularly ABC transporter genes; in contrast, PC14CDDP-miR-197 cells repressed the expression of these genes compared with that in control cells (**Supplementary Figure S2b**). Thus, the results of all of these experiments suggested that miR-197 could regulate not only drug resistance but also malignant phenotypes in lung cancer cells.

### Effects of miR-197 on tumorigenicity, pulmonary metastasis, and chemoresistance *in vivo*

**Figure 3:**
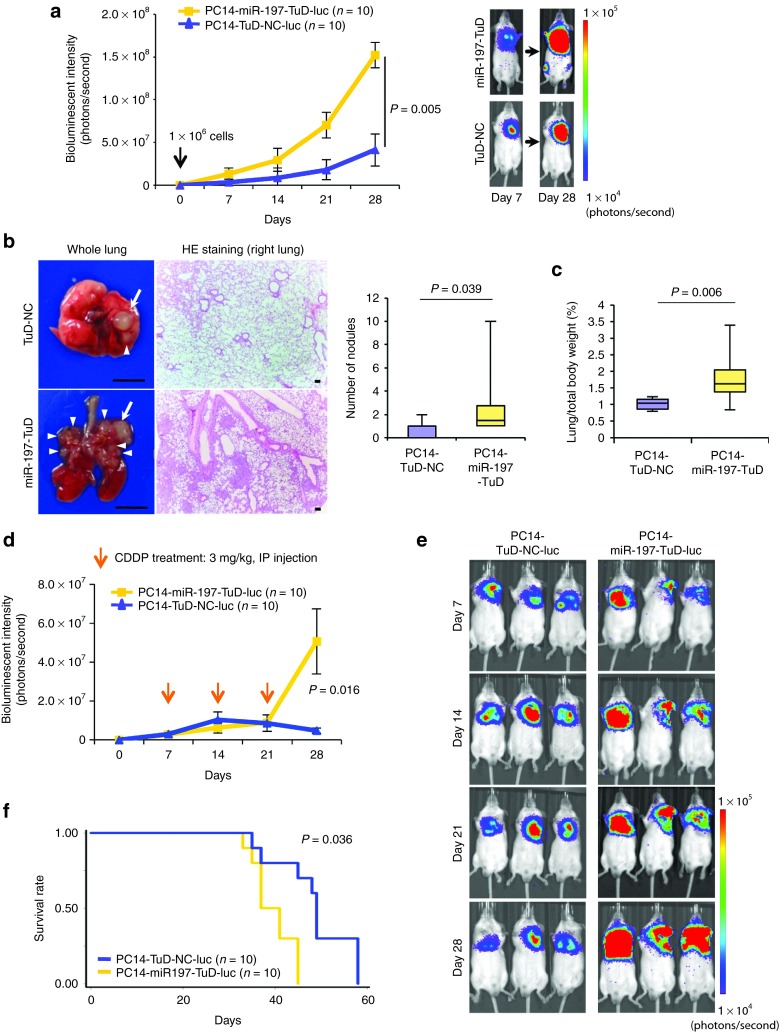
**Knockdown of miR-197 promotes tumorigenicity, pulmonary metastasis, and chemoresistance *in vivo*.** (**a**) The bioluminescent change emitted from the whole bodies of the mice bearing PC14-miR-197-TuD-luc or PC14-TuD-NC-luc cells (10 mice per group) (*P* = 0.005) (left graph) and representative bioluminescence images of lung tumor growth on days 7 and 28 (right panel). (**b**) Representative pictures of murine whole lung (left) and hematoxylin/eosin (HE) staining of the right lung are presented (right graph) (scale bar = 100 μm). Arrows indicate primary lung nodules, and triangles indicate the surface of metastatic nodules. The number of visible surface metastatic lesions in mice (10 mice per group) was significantly increased in the miR-197-attenuated group (*P* = 0.039). (**c**) The ratio of lung weights to total body weight in mice (10 mice per group) (*P* = 0.006). (**d**) The bioluminescent change emitted from the whole bodies of the mice bearing PC14-miR-197-TuD-luc or PC14-TuD-NC-luc cells (10 mice per group) after repeated intraperitoneal injections (IP injections) of CDDP (*P* = 0.016). (**e**) Representative bioluminescence images of lung tumor growth in each group after repeated administration of CDDP. (**f**) The overall survival rates in each group were estimated by the Kaplan–Meier method (*P* = 0.036). CDDP, cisplatin.

To validate the role of miR-197 as a regulator of lung cancer progression *in vivo*, we examined the effects of PC14-miR-197-TuD on tumorigenicity and chemoresistance using lung cancer xenograft models established by direct intrathoracic injection (left lung) into scid mice.^7^ To facilitate the monitoring of miRNA knockdown cells *in vivo*, we cloned PC14-miR-197-TuD or PC14-TuD-NC cells that were stably transfected with pLuc-Neo plasmid DNA. Based on bioluminescence imaging, PC14-miR-197-TuD-luc cells enhanced tumorigenic ability 28 days after implantation compared with that in negative control cell-transplanted mice (**Figure 3a**). Notably, mice-bearing PC14-miR-197-TuD-luc tumors exhibited prominent pulmonary metastases, whereas few visible metastases were observed in mice transplanted with control PC14-TuD-NC-luc cells. Histological analyses revealed that knockdown of miR-197 could promote pulmonary metastasis of this lung cancer cell line (**Figure 3b**). Furthermore, the weight of the lungs was significantly increased in the miR-197-attenuated group (**Figure 3c**). Next, to explore whether the downregulation of miR-197 affected the chemoresistance of PC14 cells to CDDP *in vivo*, tumors were initiated in scid mice, followed by the intraperitoneal injection of 3 mg/kg CDDP once a week for 3 weeks. Twenty-eight days after implantation, the tumors formed from PC14-miR-197-TuD-luc (*n* = 10) mice were significantly increased compared with those from mice-bearing PC14-TuD-NC-luc tumors (*n* = 10) on bioluminescence imaging (**Figure 3d**,**e**; *P* = 0.016). Strikingly, mice-bearing PC14-miR-197-TuD-luc tumors exhibited poor survival (**Figure 3h**; *P* = 0.036). Together, these results indicate that miR-197 plays a pivotal role in cancer progression and chemoresistance *in vivo*.

### miR-197 directly targets the cyclin-dependent kinase CKS1B

**Figure 4:**
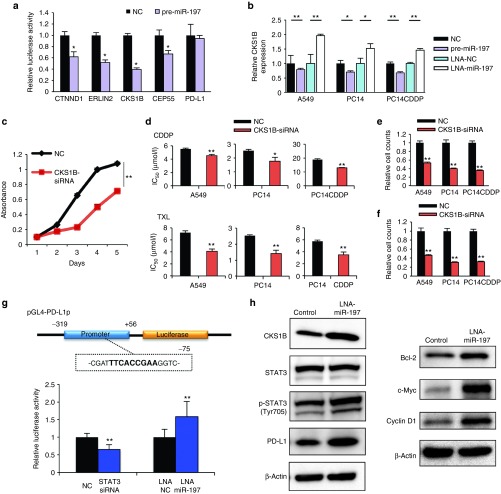
**miR-197 targets the cyclin-dependent kinase CKS1B and regulates CKS1B/STAT3 signaling.** (**a**) Dual-luciferase assays demonstrating that the repression of candidate genes by miR-197 was measured. (**b**) qRT-PCR analyses of mRNA levels of CKS1B in indicated cells treated with each miRNA. (**c**) Cell proliferation assay in A549 cells after transfection of each siRNA. (**d**) IC_50_ (CDDP or TXL) after transfection of each siRNA. (**e** and **f**) Quantification of the effect on (**e**) cell invasion and (**f**) migration after transfection of each siRNA. (**g**) The PD-L1 promoter regulatory module. A STAT3 binding site is indicated in bold. This region was cloned into a pGL-luciferase vector (pGL4-PD-L1p). Analyses of pGL4-PD-L1p luciferase activity in PC14CDDP cells treated with STAT3-siRNA, LNA-miR-197, or each NC. (**h**) Western blot analyses of target genes related to CKS1B/STAT3 signaling after treatment with LNA-miR-197 or control in PC14CDDP cells. *P < 0.05; **P < 0.01. CDDP, cisplatin; IC_50_, concentration of drug needed to inhibit cell growth by 50%; LNA, locked nucleic acid; miRNA, microRNA; mRNA, messenger RNA; NC, negative control; PD-L1, programmed cell death ligand-1; qRT-PCR, quantitative reverse transcription-PCR; siRNA, small interfering RNA; TXL, paclitaxel.

miRNAs regulate gene expression by repressing translation or promoting the degradation of their target genes. To strategically identify direct miR-197 target genes, we first performed messenger RNA (mRNA) microarray analyses of A549, PC14, and PC14CDDP cells that were transiently transfected with either pre-miR-197 or LNA-miR-197 and their controls. We observed that 1,037 predicted target genes exhibited altered expression levels consistently across the three different lung cancer cell lines (fold change ≥1.2). Next, we selected 18 highly predicted target genes using the computational algorithm TargetScan (http://www.targetscan.org) that were significantly regulated by miR-197 (average fold change of three cell lines ≥2) (**Supplementary Figure S3a**). Through qRT-PCR validation of 36 highly predicted target genes (**Supplementary Figure S3b**), we identified five candidates: catenin delta-1 (CTNND1), ER lipid raft associated 2 (ERLIN2), CDC28 protein kinase regulatory subunit 1B (CKS1B), centrosomal protein of 55 kDa (CEP55), and PD-L1. The 3′ untranslated regions (UTRs) of these genes were conjugated with luciferase for reporter assays. PD-L1 is one of the genes significantly regulated by miR-197 that does not possess the predicted binding sites in the 3′ UTR of the *in silico* database. Particularly, the 3′ UTR of CTNND1, ERLIN2, CKS1B, and CEP55 mRNA harbored sequences complementary to the miR-197 seed sequence (**Supplementary Figure S4a**). To verify whether CTNND1, ERLIN2, CKS1B, CEP55, and PD-L1 are direct targets of miR-197, we cloned each 3′ UTR into the vector downstream of luciferase open reading frame. In addition, to validate target specificity, we conducted site-directed mutagenesis of the 3′ UTR. When we cotransfected A549 cells with each of the five cloned UTRs and pre-miR-197, we observed a consistent reduction in the luciferase activity of the 3′ UTR of CTNND1, ERLIN2, CKS1B, and CEP55, but not PD-L1 (**Figure 4a**). By contrast, cotransfection of pre-miR-197 with the mutated forms of the 3′ UTRs resulted in no significant change in luciferase activity (**Supplementary Figure S4b**). Among these direct target genes, only CKS1B was reported to regulate cell motility, chemoresistance, and cancer progression.^8,9,10^ We hypothesized that CKS1B is an important target of miR-197 through its effects on chemoresistance in lung cancer. To address this hypothesis, we performed experimental validation. Inhibition of CKS1B expression by pre-miR-197 was confirmed, and the transient transfection of LNA-miR-197 significantly increased CKS1B expression in three lung cancer cell lines by qRT-PCR (**Figure 4b**). To explore whether the biological functions of miR-197 were indeed attributable to the direct targeting of CKS1B signaling, we conducted small interfering RNA (siRNA)-based silencing of CKS1B and assessed cells for cell proliferation, chemoresistance, invasion, and migration. Remarkably, we observed that siRNA-mediated knockdown of CKS1B resulted in a significant decrease in cell proliferation, invasion, and migration and sensitized the response of three lung cancer cell lines to CDDP and TXL (**Figure 4c**–**f**). These results suggest that the major function of the miR-197/CKS1B axis in NSCLC is the regulation of lung cancer progression and chemoresistance.

### PD-L1 expression is regulated by STAT3 as a key downstream target of the miR-197/CKS1B signaling pathway

Based on the analysis of the mRNA microarray, we observed that in lung cancer cells, miR-197 regulated the expression of PD-L1 (CD274, B7-H1), a member of the B7 family of costimulatory/coinhibitory ligands expressed in various tissues (**Supplementary Figure S3a**). Because of the 3′ UTR assay, we hypothesized that miR-197 might indirectly target PD-L1. Therefore, we explored the regulatory link between miR-197 and PD-L1. Previously, a constitutive oncogenic pathway has been reported to drive PD-L1 expression through signal transducer and activator of transcription 3 (STAT3) signaling in lymphoma cells.^11^ In addition, STAT3 signaling is known to be a downstream target of CKS1B activation in multiple myeloma cells.^9^ We hypothesized that CKS1B regulates PD-L1 expression by activating STAT3. Therefore, we examined the effect of modulating CKS1B on PD-L1. Consequently, siRNA-mediated knockdown of CKS1B inhibited phosphorylated (p)-STAT3 (Tyr705) and PD-L1 protein expression with no remarkable effect on STAT3 in western blots (**Supplementary Figure S5**). These data suggest that PD-L1 is an important downstream target of CKS1B activated by STAT3 in the miR-197 signaling cascade. To close the link between STAT3 and the induction of PD-L1 expression, we used PD-L1 reporter constructs containing promoter regions to determine the ability of STAT3 to bind to the PD-L1 promoter. The PD-L1 5′ sequence (−319 bp upstream to +56 bp downstream) includes a potential STAT3 binding site (TTCN_2-4_GAA)^12^ (**Figure 4g**). The cotransfection of STAT3-siRNA and PD-L1 promoter vector resulted in a 0.66 (±0.1)-fold reduction in PD-L1 promoter-driven luciferase expression compared with control siRNA. In addition, the luciferase activity was induced 1.58 (±0.4)-fold higher following LNA-miR-197 compared with that in the negative control (**Figure 4g**). Taken together, STAT3 did indeed demonstrate strong binding to the PD-L1 gene promoter downstream of miR-197 downregulation. In summary, western blots detected increased levels of CKS1B, p-STAT3, and PD-L1 with no remarkable effect on STAT3 expression downstream of miR-197 knockdown compared with that in the negative control cells. Moreover, LNA-miR-197 also induced upregulation of Bcl-2 (a representative antiapoptotic factor), c-Myc (an oncogenic transcription factor), and cyclin D1 (a cell-cycle-regulating protein), all of which are common transcriptional target genes of STAT3 (**Figure 4h**). These findings suggest that miR-197 contributes to the oncogenic activation of STAT3 *via* multiple targets through the upregulation of CKS1B.

### PD-L1 is a putative biomarker of miR-197/CKS1B/STAT3 signaling cascade

**Figure 5:**
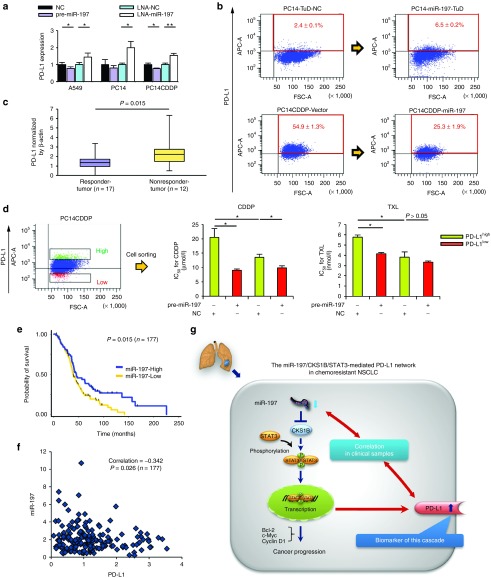
**PD-L1 is a putative biomarker regulated by the miR-197/CKS1B/STAT3 signaling cascade in NSCLC.** (**a**) qRT-PCR analyses of the mRNA levels of PD-L1 treated with each miRNA. (**b**) FCM analyses of PD-L1 expression induced by knockdown or overexpression of miR-197 in PC14 or PC14CDDP cells. (**c**) qRT-PCR analyses of mRNA levels of PD-L1 in lung tumor samples of the cohort study (*P* = 0.015). (**d**) FCM cell sorting to purify PD-L1^high^ and PD-L1^low^ cellular subsets from PC14CDDP to estimate the IC_50_ (CDDP or TXL) of PD-L1^high^ and PD-L1^low^ cells after transfection of each miRNA. (**e**) Survival outcomes of miR-197 expression in a validation cohort study (*P* = 0.015, *n* = 177). (**f**) Scatter plots between the expression of miR-197 and PD-L1 gene expression (*P* = 0.026, *n* = 177). (**g**) Scheme of the miR-197/CKS1B/STAT3-mediated PD-L1 network in chemoresistant NSCLC. The miR-197/CKS1B/STAT3 axis regulates cancer progression, and tumor PD-L1 expression as a putative biomarker of this cascade. *P < 0.05; **P < 0.01. CDDP, cisplatin; FCM, flow cytometry; IC_50_, concentration of drug needed to inhibit cell growth by 50%; miRNA, microRNA; mRNA, messenger RNA; NC, negative control; NSCLC, non-small-cell lung cancer; PD-L1, programmed cell death ligand-1; qRT-PCR, quantitative reverse transcription-PCR; TXL, paclitaxel.

The recent launch of an anti-PD-L1 antibody has represented a significant breakthrough for patients with advanced solid tumors.^4^ Strikingly, blockade of PD-L1 induced durable tumor regression and prolonged disease stabilization in patients with advanced NSCLC. To explore the role of PD-L1 in the miR-197-mediated signaling pathway in NSCLC, we analyzed the expression of PD-L1 in lung cancer cell lines and lung cancer samples. The specific regulation of PD-L1 expression by miR-197 was validated by qRT-PCR analysis, demonstrating both a significant inhibitory effect of pre-miR-197 in A549 and PC14CDDP cells and a stimulating effect of LNA-miR-197 on PD-L1 expression in three lung cancer cell lines (**Figure 5a**). Flow cytometry (FCM) analysis revealed that the level of PD-L1 in PC14CDDP cells was significantly higher than that in the parental PC14 cells (**Supplementary Figure S6**). Moreover, the expression of PD-L1 in the PC14 cells was significantly upregulated after the knockdown of miR-197. Conversely, the overexpression of miR-197 induced decreased expression of PD-L1 in the PC14CDDP cells in FCM analysis (**Figure 5b**). To further examine whether PD-L1 is a candidate chemoresistant marker for NSCLC samples, we compared the mRNA level of PD-L1 expression in the responder group (*n* = 17) with that in the nonresponder group (*n* = 12) of the cohort study. We also observed increased expression of PD-L1 in the tumors of the nonresponder group compared with that in the tumors of the responder group (**Figure 5c**; *P* = 0.015). Next, we isolated PD-L1^high^ and PD-L1^low^ cells from PC14CDDP by FCM cell sorting to examine whether the PD-L1^high^ fraction might be associated with chemoresistance regulated by miR-197-mediated signaling. The PD-L1^high^ cells demonstrated significant resistance to CDDP and TXL compared with the PD-L1^low^ cells (**Figure 5d**). The transient transfection of pre-miR-197 sensitized the response of PD-L1^high^ cells to CDDP and TXL. Furthermore, the transfection sensitized the response of PD-L1^low^ cells to CDDP (**Figure 5d**). These data suggest that the miRNA-197-mediated network can drive PD-L1 expression as a surrogate marker of this signaling cascade.

### The importance of miR-197 and PD-L1 in a clinically relevant pathway

To further understand the potential biological significance of downregulated miR-197 expression in lung cancer progression, we evaluated the correlation of the miR-197 expression profile with overall survival in tumor specimens from 177 NSCLC patients who experienced recurrence after curative resection (**Supplementary Table S2**). The levels of miR-197 were measured by qRT-PCR, and Kaplan–Meier analysis indicated that lower expression levels of miR-197 were significantly associated with worse overall survival (**Figure 5e**; *P* = 0.015). Next, we examined the association of miR-197 with PD-L1, a putative biomarker of this signaling. Based on tumor data with combined mRNA and miRNA expression profiles, we observed a negative correlation between miR-197 and PD-L1 expression (**Figure 5f**; *P* = 0.026). The above data suggest a signaling network that links miR-197 with its downstream target PD-L1 and demonstrates the clinical importance of the miR-197-regulated pathway in NSCLC (**Figure 5g**).

## Discussion

Here, we identified that miR-197 is downregulated in lung cancer tissues in patients resistant to platinum-based chemotherapy after recurrence. We further demonstrated that a novel signaling pathway, the miR-197/CKS1B/STAT3 axis, has the ability to promote cancer progression in chemoresistant NSCLC. Previous evidence has indicated that miR-197 is dysregulated in lung cancer,^13^ thyroid cancer,^14^ tongue cancer,^15^ and pancreatic cancer,^16^ strongly suggesting a general function of miR-197 in different types of tumors. Moreover, other researchers have reported that the downregulation of miR-197 has the potential to contribute to multidrug resistance in colon cancer cells^17^ and head and neck cancer cells,^18^ implying that miR-197 plays an essential role in regulating several signaling pathways, including tumor progression and chemoresistance. Our data demonstrated that miR-197 regulates lung cancer drug resistance and tumor progression by directly targeting the cyclin-dependent kinase CKS1B as well as by indirectly targeting the transcription factor STAT3. Notably, we discovered that the CKS1B/STAT3 axis is the most powerful signaling pathway regulated by miR-197 because of the activation of various oncogenic target genes, including Bcl-2, c-Myc, and cyclin D1. Recently, it has been reported that STAT3 signaling has an important role in tumor initiation in various types of cancers.^19^ Therefore, phosphorylation of STAT3 induced by miR-197/CKS1B signaling may also promote cancer stem cell phenotypes in NSCLC.

In this study, we demonstrated that the miR-197/CKS1B/STAT3-mediated signaling drives tumor PD-L1 expression, allowing it to function as a biomarker for this cascade in NSCLC. PD-L1 is a T-cell coinhibitory molecule expressed in various types of cancers that leads to the immune escape of tumor cells.^5^ Indeed, previous reports have suggested that PD-L1 protein expression might be associated with worse prognoses in NSCLC.^20^ Recent strategies targeting the interaction of PD-L1 with its receptor, programmed cell death-1 (PD-1), have demonstrated tremendous efficacy in early phase clinical trials enrolling NSCLC patients.^4^ Although many studies have focused on the relationship between PD-L1 and immune escape, there are limited data on the prevalence and prognostic role of PD-L1 expression in NSCLC cells. Moreover, it has been demonstrated that PD-L1 expression is driven by constitutive oncogenic signaling pathways in some tumors, such as glioblastomas.^21^ However, it is unclear whether a specific upstream signaling pathway of PD-L1 independent of immunoinhibitory signals in NSCLC. Our data suggest that PD-L1-targeted therapy might be a reasonable therapeutic strategy for NSCLC because PD-L1 is regulated by the miR-197/CKS1B/STAT3-mediated signaling cascade. In addition, we observed a significant correlation between miR-197 and PD-L1 expression in a validated cohort study (*n* = 177, *P* = 0.026). Presently, the evaluation of PD-L1 positivity by conventional immunohistochemistry is not well defined.^22^ Therefore, the correlation between miR-197 and PD-L1 also suggests that miR-197 expression can act as a surrogate biomarker of PD-L1 in NSCLC.

Consistent with our finding that miR-197 can regulate chemoresistance, our results further suggested that PD-L1 is a biomarker of the miR-197/CKS1B/STAT3-mediated cascade in NSCLC. Although it has been recently reported that PD-L1 expression is enhanced after cytotoxic drug treatment in breast cancer^23^ and melanoma cells,^24^ the mechanisms of biological interaction between PD-L1 and chemoresistance are unclear. In the analyses of FCM cell sorting, we found that the PD-L1^high^ lung cancer cells exhibited significant resistance to CDDP and TXL compared with that in the PD-L1^low^ cells. Moreover, we observed increased expression of PD-L1 in the tumors of the nonresponder group compared with that in the responder group. In the current study, we also demonstrated that the miR-197 mimic sensitized PD-L1^high^ cells to cytotoxic chemotherapy. These data suggest that miR-197 replacement therapy may be an effective treatment for lung cancer patients with chemoresistance, particularly in PD-L1-positive patients.

Exploration of the regulatory mechanisms of abnormal miRNAs expressed in lung cancer is of great interest. In this study, we have not identified the transcriptional regulators of miR-197. This miRNA is located in the chromosomal 1p13 region. Loss of heterozygosity of chromosome arm 1p is common in lung cancer and other cancers.^25^ It has been reported that loss of heterozygosity at this region occurs in over 45% of tobacco smokers with squamous cell carcinoma and adenocarcinoma.^26^ In our cohort study for miRNA microarray, the population of smokers was high, with 88% in the responder group and 67% in the nonresponder group. Remarkably, the glutathione *S*-transferases Mu (GSTM) gene clusters are also located in this region.^27^ GSTs play an important role in cellular defense because they are involved in the detoxification of many carcinogens from smoking and facilitate their excretion. A reported 38 and 67% of Caucasians carry a deletion in both alleles of the glutathione *S*-transferases M1 (GSTM1) gene, resulting in the total absence of GSTM1 enzyme activity; the GSTM1 null genotype confers an increased risk of lung cancer.^28,29^ To date, we have considered that genetic changes such as gene deletions and loss of heterozygosity in this region might induce smoking-induced lung cancer development and downregulation of miR-197. In addition to transcriptional regulation, other mechanisms could contribute to deregulated miRNA expression, including chromatin deletions/amplifications, translocations, DNA methylation, promoter silencing, and deficient posttranscriptional processing.

In conclusion, we have identified a novel signaling network, the miR-197/CKS1B/STAT3 axis, which regulates cancer progression in chemoresistant NSCLC. Moreover, we demonstrated that the miR-197-mediated pathway drives tumor PD-L1 expression as a putative biomarker of this cascade, independent of immunoinhibitory activities. These data suggest novel insights regarding the therapeutic potential of miRNA-197 for chemoresistant NSCLC, particularly in PD-L1-positive patients. The molecular network underlying the development of tumor malignancy may open up new avenues for cancer therapeutics and biomarkers in various solid tumors.

## Materials and Methods

***Clinical lung cancer samples.*** The study protocol was approved by the Institutional Review Board at the National Cancer Center (Number: 2012–054). All materials were obtained with written informed consent and were provided by the National Cancer Center Biobank. All human lung tissue samples were derived from the resected lungs of lung cancer patients who had recurrence after pulmonary resection at the Department of Thoracic Surgery in the National Cancer Center Hospital between 1997 and 2010. According to the clinical database obtained from the Department of Thoracic Oncology Division in National Cancer Center Hospital, we selected 29 lung tumor samples with corresponding normal lung tissues for the miRNA microarray analysis and 177 lung tumor samples for validated cohort study (**Supplementary Tables S1** and **S2**). In this study, the patients who were diagnosed with NSCLC (adenocarcinoma or squamous cell carcinoma) were enrolled. Upon removal of the surgical specimen, the research person immediately transported the tissues to the Biobank. Tissues were stored at −80 °C after snap freezing in liquid nitrogen. All tumors were reviewed by pathologists and were pathologically diagnosed according to the World Health Organization Classification of Tumors.

***Chemotherapeutic response in clinical lung cancer samples.*** The chemotherapeutic response of tumors for platinum-based chemotherapy was clinically evaluated according to the Response Evaluation Criteria In Solid Tumors (RECIST) as follows: (i) complete response, disappearance of all known disease; (ii) partial response, 30% or more decrease in the entire tumor burden; (iii) stable disease, less than 30% decrease or less than 20% increase in the entire tumor burden; and (iv) progressive disease, 20% increase in the entire tumor burden or appearance of new lesions. In our report, subjects with complete response and partial response were defined as responders, and subjects with progressive disease were defined as nonresponders. Platinum-based chemotherapy was given for four or more cycles after recurrence. No cases received adjuvant chemotherapy after curative resection.

***Cell culture.*** Human lung cancer cell lines A549 (lung adenocarcinoma), PC9 (lung adenocarcinoma), PC14 (lung adenocarcinoma), H226 (lung squamous cell carcinoma), H358 (lung adenocarcinoma), and human bronchial epithelial cell line BEAS-2B were purchased from ATCC (Manassas, VA). PC9CDDP (lung adenocarcinoma) and PC14CDDP (lung adenocarcinoma)^30^ were provided by Shien-Lab, Medical Oncology, National Cancer Center Hospital of Japan. These cells are maintained in RPMI 1640 medium with 10% heat-inactivated fetal bovine serum and an antibiotic-antimycotic (Invitrogen, Carlsbad, CA) at 37 °C in 5% CO_2_. The cumulative culture length of the cells was fewer than 6 months after resuscitation. Early passage cells were used for all experiments and they were not reauthenticated.

***RNA extraction, RT, qRT-PCR, and microarray analysis.*** Total RNA was extracted from cultured cells or human lungs using QIAzol and the miRNeasy Mini Kit (Qiagen, Hilden, Germany) according to the manufacturer's protocol. All clinical samples exhibited RNA integrity numbers >6.0. The qRT-PCR method has been previously described.^31^ Hsa-miR-197, endogenous control RNU6B, RNU44 TaqMan qRT-PCR kits, human-β-actin, and the 36 predicted target genes' TaqMan Gene Expression Assays were purchased from Applied Biosystems (Foster City, CA). SYBR Green I qRT-PCR was performed, and the β-actin housekeeping gene was used to normalize the variation in the complementary DNA levels. All primer sequences are listed in **Supplementary Table S3**. To detect the miRNAs in lung tissues for microarray, 500 ng of total RNA was labeled and hybridized using 3D-Gene miRNA Oligo chip (Ver. 17.0; Toray, Tokyo, Japan) according to the manufacturer's protocol. Hybridization signals were detected using a 3D-Gene Scanner 3000 (Toray), and the scanned images were analyzed using 3D-Gene extraction software (Toray) (Accession No. GSE56036). To detect the mRNAs in cells, 100 ng of total RNA was labeled and hybridized using a SurePrint G3 Human GE v2 8x60K Microarray (Agilent Technologies, Santa Clara, CA) according to the manufacturer's protocol. Hybridization signals were detected using a DNA microarray scanner (Agilent Technologies) (Accession No. GSE56266).

***Cell viability assay.*** A Cell Counting Kit-8 (CCK-8) (Dojindo Laboratories, Kumamoto, Japan) was used in the cell viability assay. Three thousand cells per well were seeded in 96-well plates. The following day, the cells were replenished with fresh medium containing 25 nmol/l of each miRNA or siRNA. The cells were used for cell proliferation assays or cytotoxicity assays. The absorbance at 450 nm was measured using Envision (PerkinElmer, Norwalk, CT). The IC_50_ (concentration of drug needed to inhibit cell growth by 50%) is generated from the dose–response curves for each cell line.

***Cell invasion and migration assays.*** For the transwell migration assay, 50,000 cells were placed in the top chamber of each insert (8 μm pore size; BD Biosciences, Franklin Lakes, NJ) without matrigel coating. For the invasion assay, 1 × 10^5^ cells were placed on the upper chamber of each insert in 24-well Biocoat Matrigel invasion chambers (8 μm pore size; BD Biosciences). The cells that migrated through the membrane and adhered to the lower surface of the membrane were fixed with methanol and stained with Diff Quick staining. For quantification, the cells were counted under a microscope in four random fields.

***Immunoblot analysis.*** Sodium dodecyl sulfate–polyacrylamide gel electrophoresis gels were calibrated using Precision Plus protein standards (161–0375) (Bio-Rad, Hercules, CA) and anti-CKS1B (1:200), anti-STAT3 (1:1,000), anti-p-STAT3 (1:2,000), anti-PD-L1 (1:1,000), anti-Bcl-2 (1:200), anti-c-Myc (1:1,000), anti-Cyclin D1 (1:200), and anti-actin (1:1,000) were used as the primary antibodies. Two secondary antibodies (peroxidase-labeled anti-mouse and anti-rabbit antibodies) were used at a dilution of 1:10,000.

***FCM analysis and cell sorting.*** Cells were suspended in their culture medium and subjected to a FACSAria II cell sorter (BD Biosciences) for FCM analysis and cell sorting. At least one million cells were pelleted by centrifugation at 180*g* for 5 minutes at 4 °C, resuspended in 20 μl of a monoclonal mouse antihuman PD-L1-APC antibody (eBioscience, San Diego, CA) or a monoclonal mouse IgG1 K isotype control APC antibody (eBioscience) solution, and incubated for 30 minutes at 4 °C.

***Animal studies.*** Animal experiments were performed in compliance with the guidelines of the Institute for Laboratory Animal Research, National Cancer Center Research Institute (Number: T12-004); 6–7 weeks old male C.B-17/Icr-scid/scidJcl mice (CLEA, Tokyo, Japan) were used in the experiments. Intrathoracic injections were performed following a previous protocol with minor modifications.^7^ For *in vivo* imaging, the mice were administered 150 mg/kg D-luciferin (Promega, Madison, WI) by intraperitoneal injection. Ten minutes later, photons from the whole bodies of the animals were counted by measuring bioluminescence with an IVIS Spectrum imaging system (Caliper Life Science, Hopkinton, MA). The data were analyzed using LIVINGIMAGE 4.2 software (Caliper Life Science). The development of lung cancer was monitored twice a week *in vivo* by bioluminescent imaging. In the repeated administration study, the treatment (3 mg/kg of CDDP) was performed on days 7, 14, and 21 (once a week for 3 weeks, three treatments total).

***Statistical analyses.*** All experiments were repeated at least three times, and the results are expressed as the means ± SD. The statistical analyses were mainly conducted using Student's *t*-test. The analysis of miRNA microarrays for clinical samples was conducted using analysis of variance. Pearson's correlation was estimated between miR-197 and PD-L1. The Kaplan–Meier method was used to estimate survival as a function of time, and survival differences were analyzed by the log-rank test. All of the analyses were performed using PASW statistics version 18.0 (IBM, Armonk, NY) and STATA version 12.0 (Stata Corp, College Station, TX). *P* values <0.05 indicated a statistically significant difference.

A detailed description of the materials and methods used in this study are found in **Supplementary Materials and Methods**.

**SUPPLEMENTARY MATERIAL** **Figure S1**. Statistical analysis of the miRNA microarray in a cohort study. **Figure S2**. miR-197 regulates malignant phenotypes of lung cancer cells *in vitro*. **Figure S3**. Prediction of target candidates for miR-197. **Figure S4**. CTNND1, ERLIN2, CKS1B, and CEP55 are direct targets of miR-197. **Figure S5**. Western blot analyses of the downstream targets of CKS1B. **Figure S6**. FCM analyses of the PD-L1 levels of A549, PC14, and PC14CDDP. **Table S1**. Characteristics of participating patients of a cohort study (*n* = 29). **Table S2**. Characteristics of participating patients of a validation cohort study (*n* = 177). **Table S3**. The primer sequences for qRT-PCR analyses. **Materials and Methods**
