# Supplementary material for: The Clinical Relevance of the miR-197/CKS1B/STAT3-mediated PD-L1 Network in Chemoresistant Non-small-cell Lung Cancer
Source: Mol Ther. 2015 Feb 10;23(4):717–27. doi: 10.1038/mt.2015.10 (PMC4395779; doi:10.1038/mt.2015.10)
Supplement: Supplementary Figures and Tables [file mt201510x1.pdf]

Supplementary Materials

The clinical relevance of the miR-197/CKS1B/STAT3-mediated PD-L1 network in chemoresistant non-small-cell lung cancer

Yu Fujita, Shigehiro Yagishita, Keitaro Hagiwara,
Yusuke Yoshioka, Nobuyoshi Kosaka, Fumitaka Takeshita,
Tomohiro Fujiwara, Koji Tsuta, Hiroshi Nokihara,
Tomohide Tamura, Hisao Asamura, Makoto Kawaishi,
Kazuyoshi Kuwano, and Takahiro Ochiya

Supplementary Figure S1-S6
Supplementary Table S1-S3

Supplementary Figure S1. Statistical analysis of the miRNA microarray in a cohort study. (A) As identified by ANOVA analysis of 4 groups, 17 miRNAs (6 upregulated miRNAs and 11 downregulated miRNAs in non-responder's tumors) exhibited significant expression changes. (B) As identified by quantitative RT-PCR (qRT-PCR) of tumor samples from the two groups, 5 miRNAs (miR-197, miR-181c, miR-21, miR-210, and miR-1260) exhibited significant expression changes. (C) Overall survival Kaplan-Meier curves were based on miR-197 or miR-1260 expression; median miRNA expression was used to define low and high expressers (miR-197; p=0.014, miR-1260; p=0.037).

Supplementary Figure S2. miR-197 regulates malignant phenotypes of lung cancer cells *in vitro*. (A) Representative images (upper panels, pictures) and quantification (lower panels, graphs) of indicated cells grown using the 3D spheroid assay (scale bar 100 mm). (B) qRT-PCR analysis of the expression of various lung cancer initiation-related genes in PC14-miR-197-TuD and PC14CDDP-miR-197 cells compared with that in each control cell line. Experiments were repeated at least three times with similar results, and error bars represent the mean ± SD, *p<0.05. **p< 0.01. NC, negative control.

Supplementary Figure S3. Prediction of target candidates for miR-197. (A) mRNA microarray analyses of A549, PC14, and PC14CDDP cells that were transiently transfected with either pre-miR-197 or LNA-miR-197 and their controls. We observed that 1037 predicted target genes exhibited altered expression consistently across the three different lung cancer cell lines (fold change ≥ 1.2). We used two algorithms with mRNA microarray data and the in silico database for prediction analyses to identify target genes of miR-197. (B) Through qRT-PCR validation of 36 highly predicted target genes, five candidates (CTNND1, ERLIN2, CKS1B, CEP55, and PD-L1) were discovered. NC, negative control.

Supplementary Figure S4. CTNND1, ERLIN2, CKS1B, and CEP55 are direct targets of miR-197. (*A*) Schematic miR-197 putative target sites in the 3' UTRs of CTNND1, ERLIN2, CKS1B, and CEP55 and the sequence of mutant UTRs. (*B*) The effect of co-transfection of pre-miR-197 with wild-type (Wt) and mutant (Mut) psiCHECK2 vectors with each gene construct in A549 cells was measured by luciferase reporter assays. Experiments were repeated at least three times with similar results, and error bars represent the mean ± SD. *$p<0.05$. NC, negative control.

Supplementary Figure S5. Western blot analyses of the downstream targets of CKS1B. siRNA-mediated knockdown of CKS1B inhibited phosphorylated (p)-STAT3 (Tyr705) and PD-L1 protein expression with no remarkable effect on STAT3 in western blots using β-actin as an internal control. Experiments were repeated at least three times with similar results.

Supplementary Figure S6. FCM analyses of the PD-L1 levels of A549, PC14, and PC14CDDP. The PD-L1$^+$ cell level analyses by FCM of A549 (5.5 ± 0.2%), PC14 (2.6 ± 0.3%), and PC14CDDP (51.8 ± 1.2%) (mean ± SD). Experiments were repeated at least three times with similar results.

Supplementary Figure S1

A

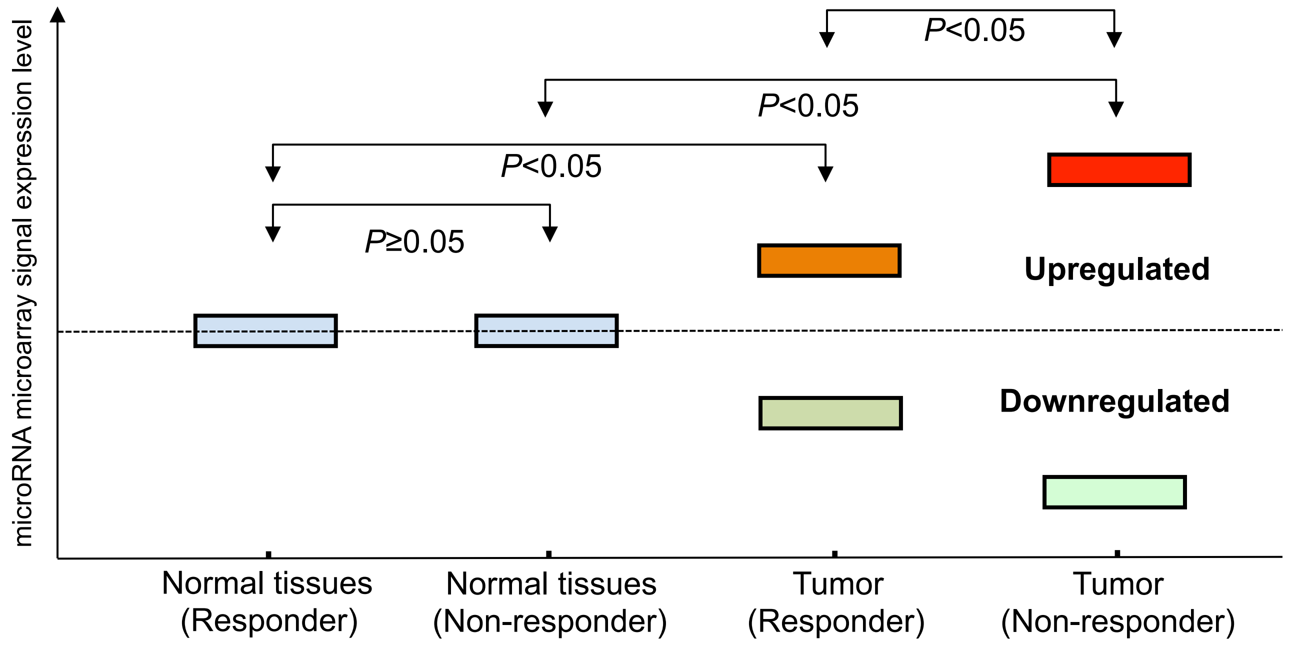

ANOVA

Upregulated miRNAs in non-responder's tumors;
hsa-miR-21, hsa-miR-142-3p, hsa-miR-181c, hsa-miR-182, hsa-miR-210, hsa-miR-1260

Downregulated miRNAs in non-responder's tumors;
hsa-miR-29c, hsa-miR-30a*, hsa-miR-30e*, hsa-miR-126, hsa-miR-139-5p, hsa-miR-144, hsa-miR-197, hsa-miR-338-3p, hsa-miR-497, hsa-miR-663b, hsa-miR-1231

qRT-PCR validation of tumor samples
(17 miRNAs)

Supplementary Figure S1

B

qRT-PCR validation of tumor samples (17 miRNAs)

1) qRT-PCR validation of upregulated miRNAs in non-responder's tumor

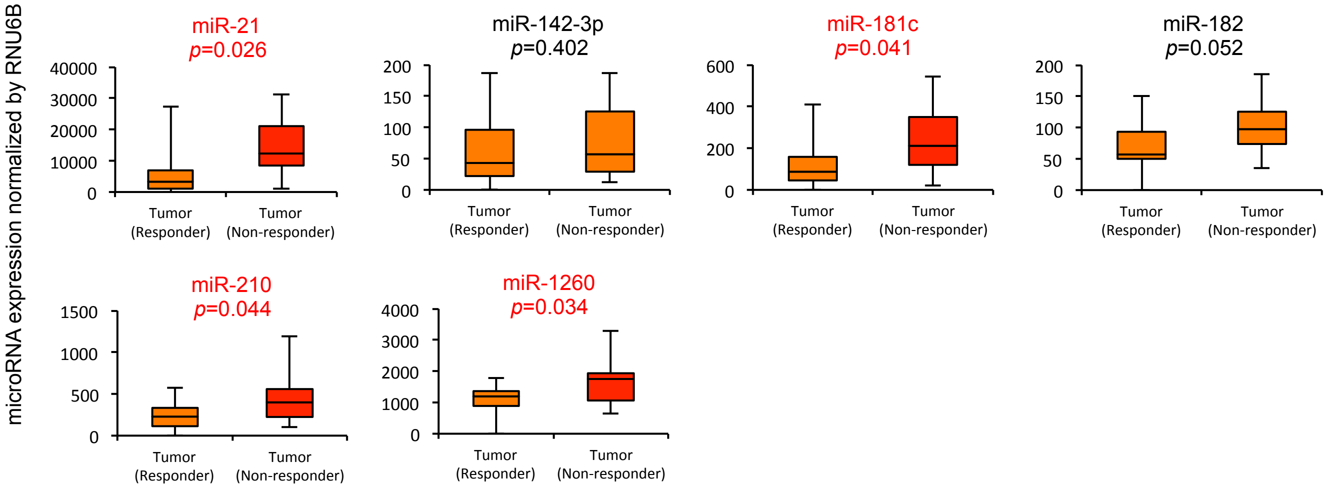

2) qRT-PCR validation of downregulated miRNAs in non-responder's tumor

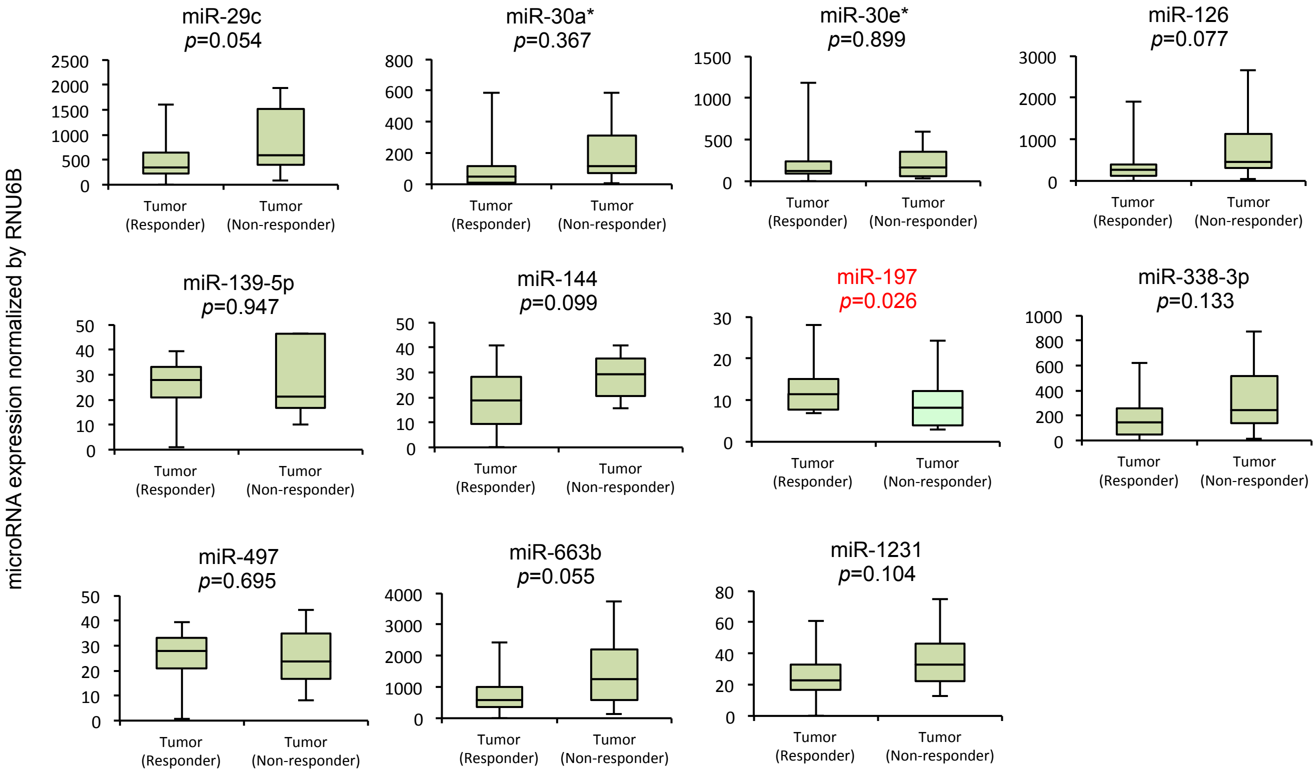

5 miRNAs related with drug resistance in this cohort study;
hsa-miR-21, hsa-miR-181c, hsa-miR-210, hsa-miR-1260, hsa-miR-197

Supplementary Figure S1

C

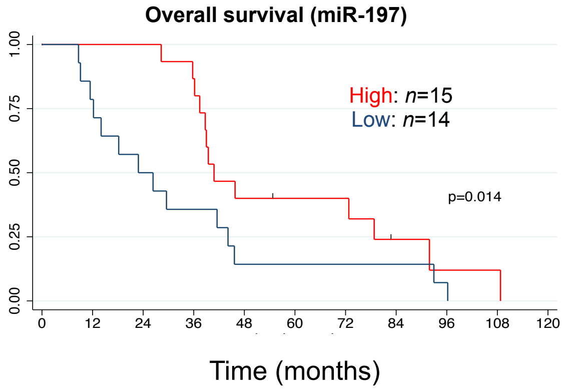

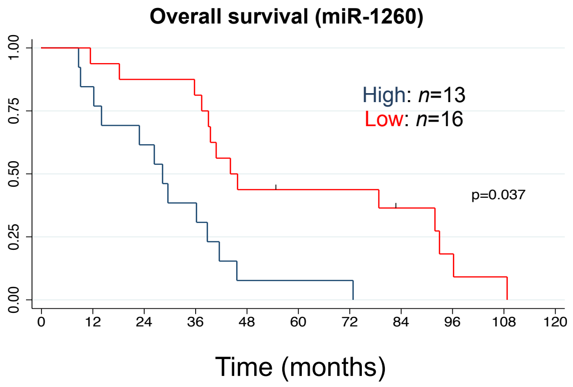

Supplementary Figure S2

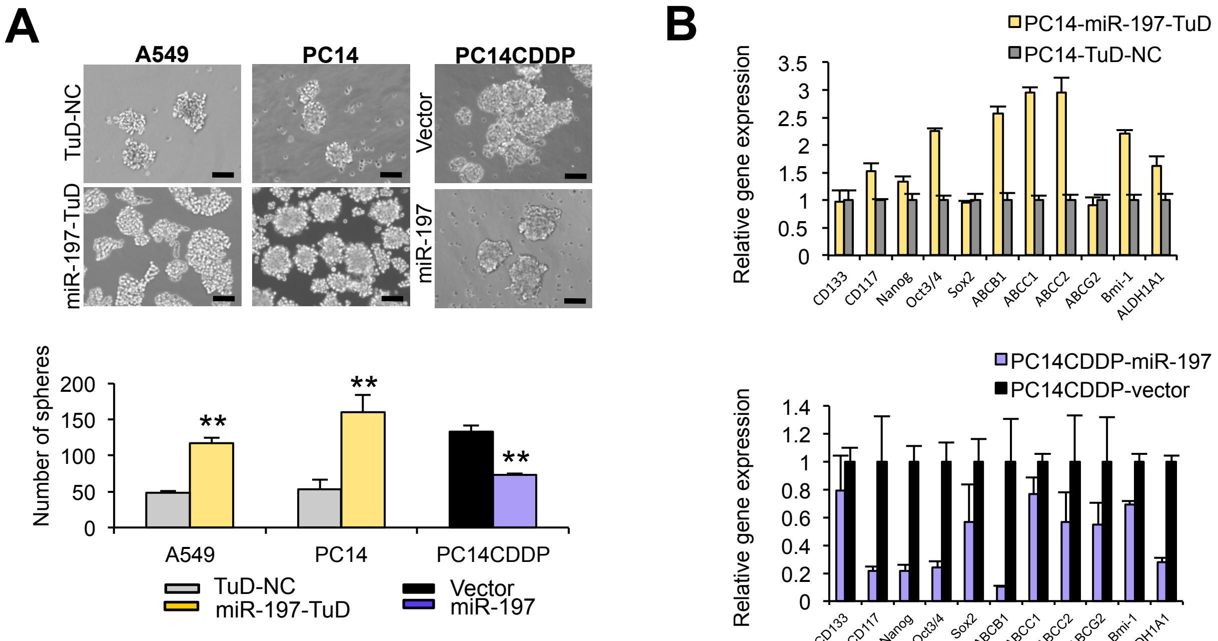

Supplementary Figure S3

A

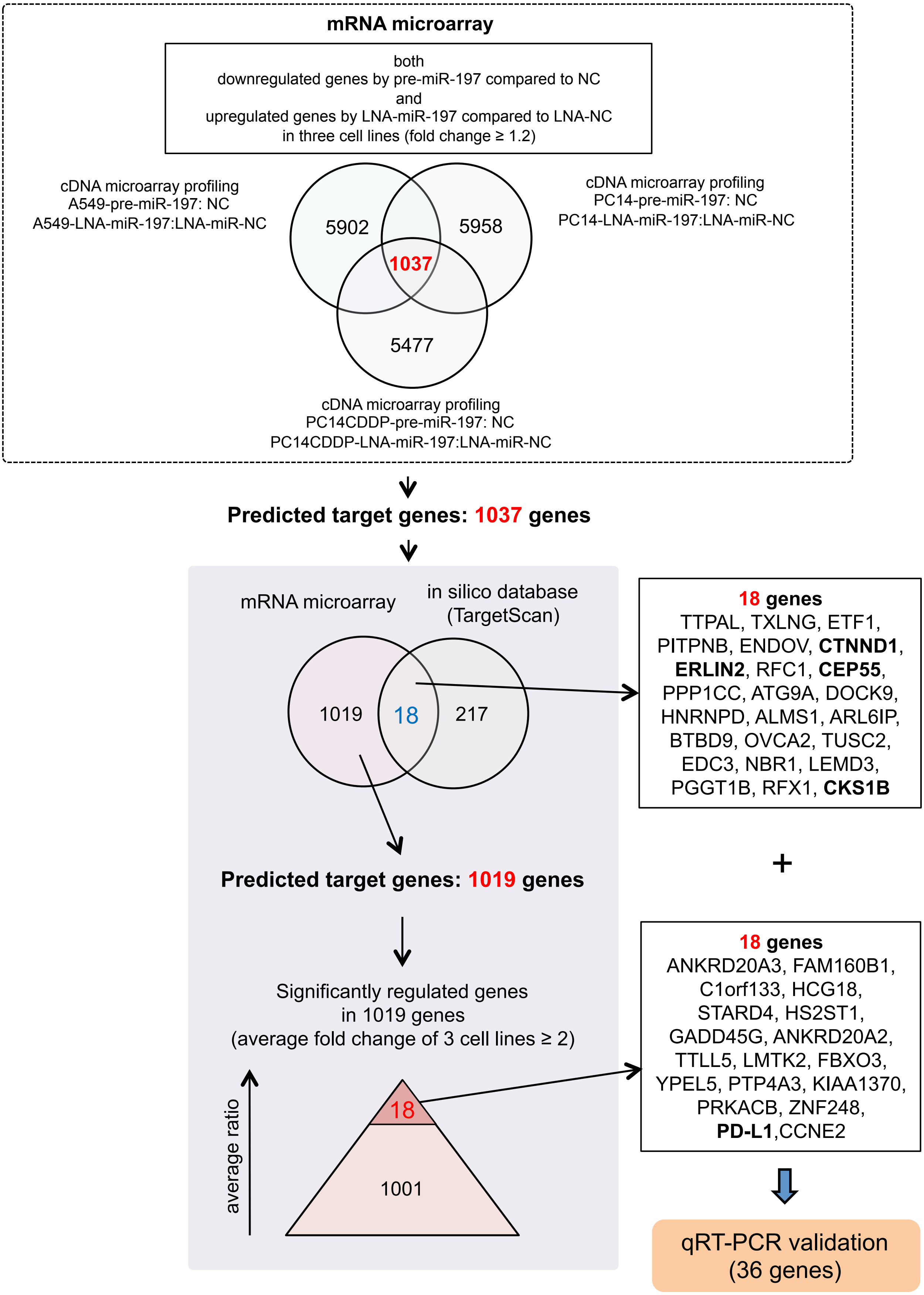

Supplementary Figure S3

B

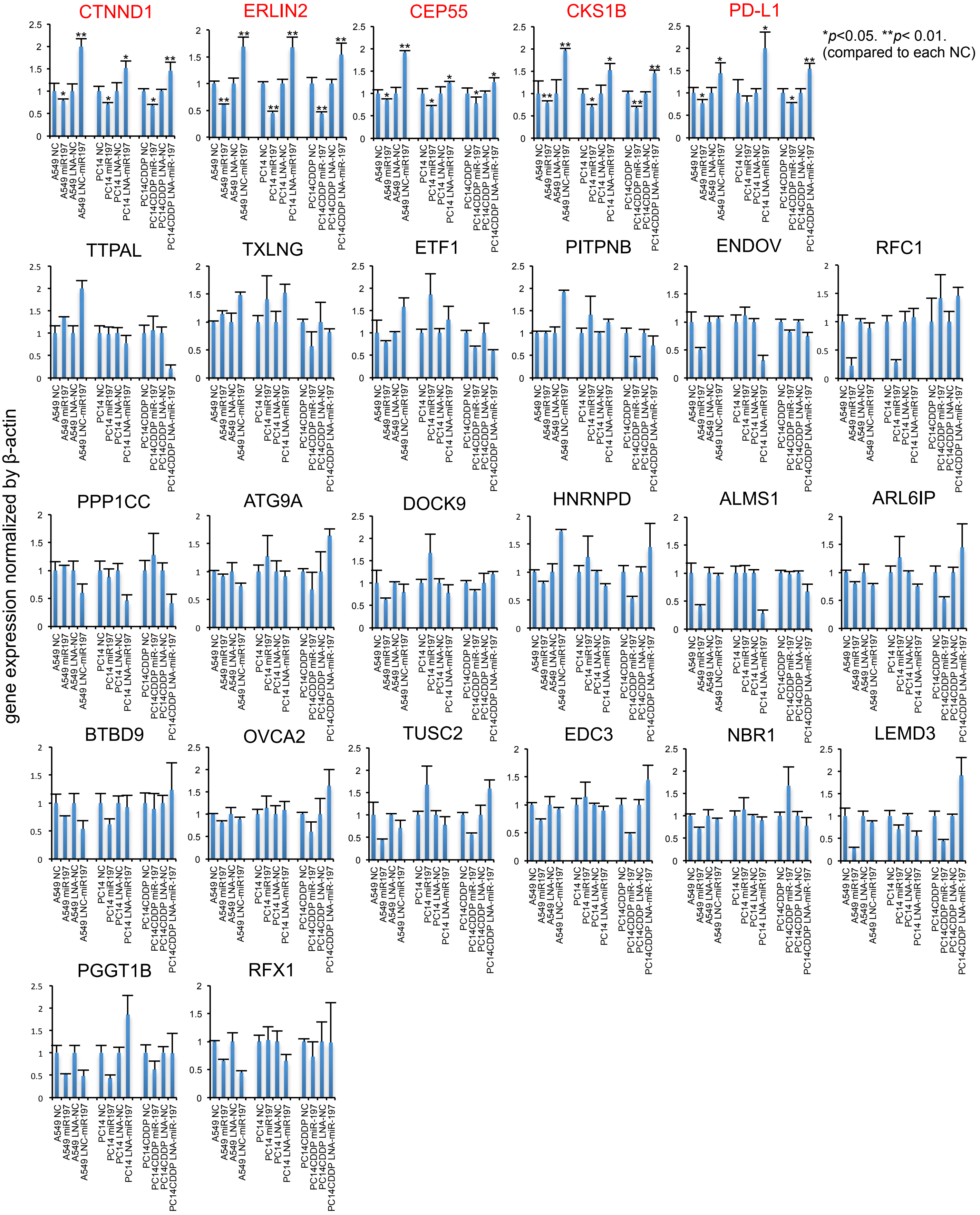

Supplementary Figure S3

B (cont)

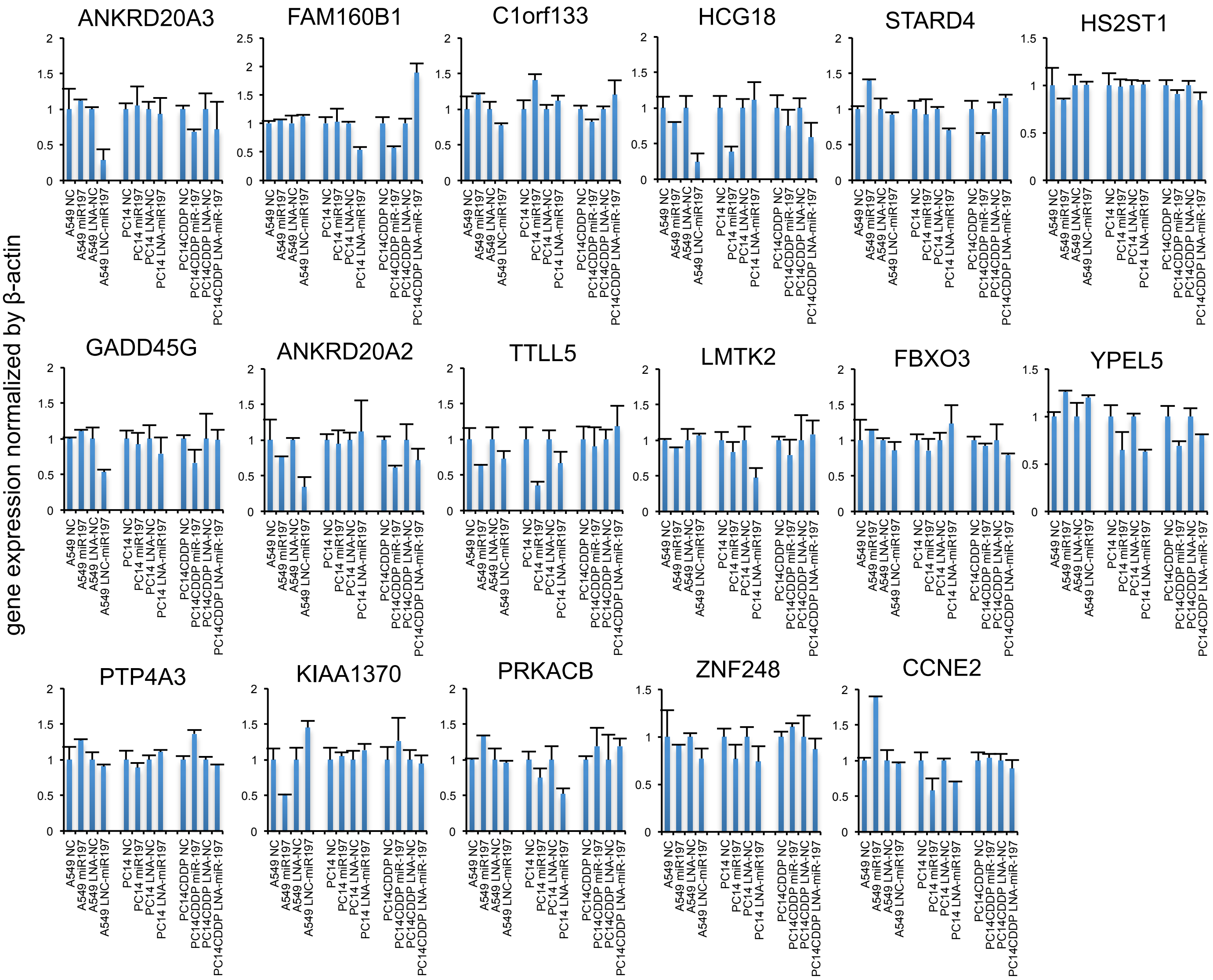

The selection criteria of predicted target genes:
both
downregulated genes by pre-miR-197 compared to NC
and
upregulated genes by LNA-miR-197 compared to LNA-NC
in three cell lines by qRT-PCR

<u>Prediced target genes</u>
→CTNND1, ERLIN2, CKS1B, CEP55, PD-L1

Supplementary Figure S4

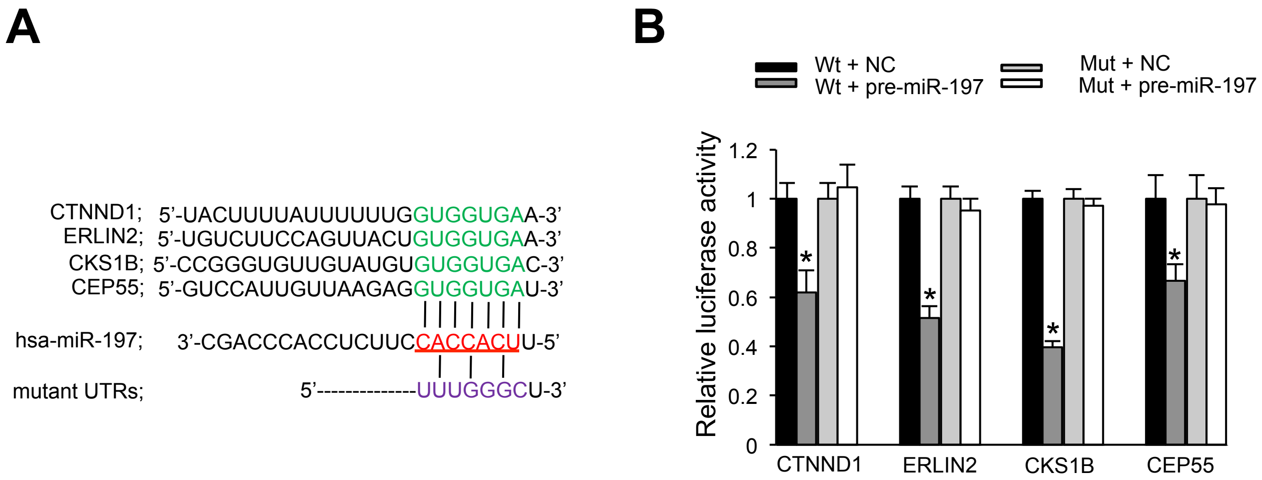

Supplementary Figure S5

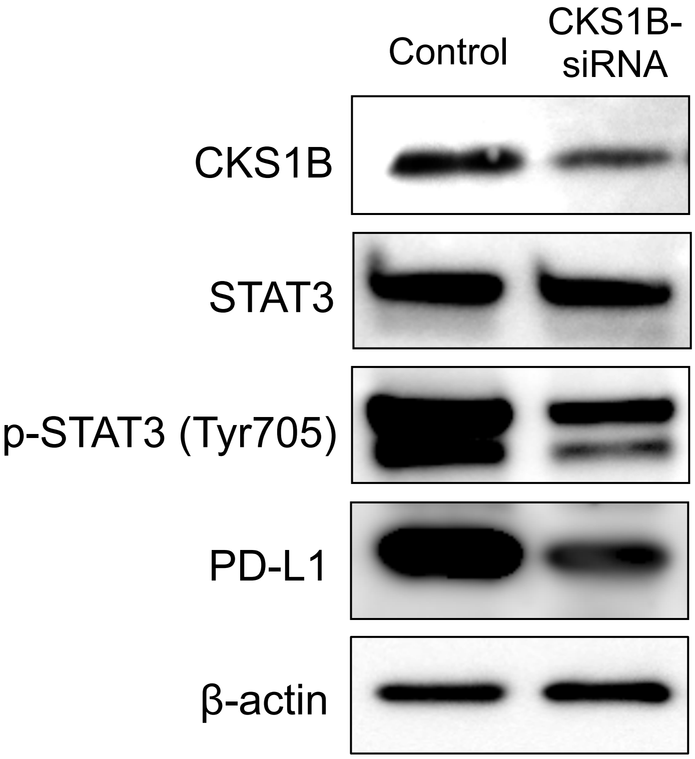

Supplementary Figure S6

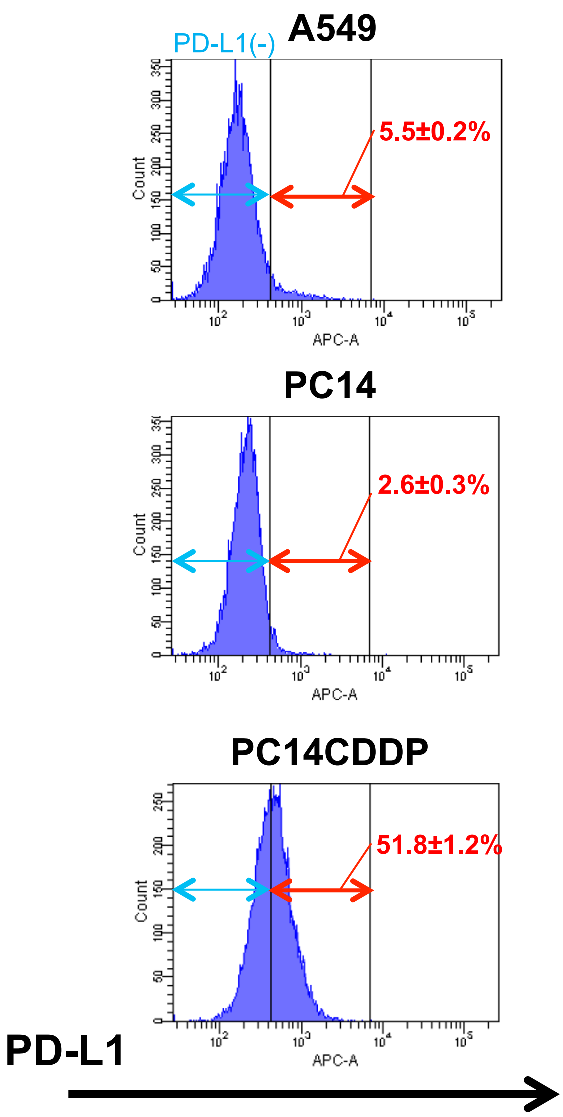

Supplementary Table S1. Characteristics of participating patients of a cohort study (*n*=29)

Patient and Disease Characteristics		
	**Responder group**	**Non-responder group**
**Number of patients**	*n*=17	*n*=12
Median Age (Range)	62 (44-74)	67 (46-74)
Gender (%)		
Male / Female	13 / 4 (77 / 23)	10 / 2 (83 / 17)
Histological subtype (%)		
Adenocarcinoma	9 (53)	9 (75)
Squamous cell carcinoma	8 (47)	3 (25)
Smoking status (%)		
Never / Former or Current	2 / 15 (12 / 88)	4 / 8 (33 / 67)
Pathological Stage (%)		
Stage I	2 (12)	5 (42)
Stage II	6 (35)	1 (8)
Stage III	9 (53)	6 (50)
Stage IV	0 (0)	0 (0)
Chemotherapy regimen(%)		
Carboplatin-based	15 (88)	9 (75)
Cisplatin-based	2 (12)	3 (25)

Supplementary Table S2. Characteristics of participating patients of a validation cohort study (*n*=177)

Patient and Disease Characteristics	
	**Validation cohort**
**Number of patients**	*n*=177
Median Age (Range)	65 (34-82)
Gender (%)	
Male / Female	111 / 66 (63 / 37)
Histological subtype (%)	
Adenocarcinoma	151 (85)
Squamous cell carcinoma	26 (15)
Smoking status (%)	
Never / Former or Current	67 / 110 (38 / 62)
Pathological Stage (%)	
Stage I	57 (32)
Stage II	23 (13)
Stage III	97 (55)
Stage IV	0 (0)

Supplementary Table S3. The primer sequences for qRT-PCR analyses

Gene	Forward primer (5'-Sequence-3')	Reverse primer (5'-Sequence-3')
CD133	ggacccattggcattctc	caggacacagcatagaataatc
CD117	ggcgcgagggagggggaggcgagga	aagtgcagcgagcgcggcaaagcc
Nanog	cagtctggacactggctgaa	ctcgctgattaggctccaac
OCT3/4	agtgagaggcaacctggaga	acactcggaccacatccttc
SOX2	ggagctttgcaggaagtttg	tttttcgtcgcttggagact
ABCB1	cttatgctctggccttctgg	tgcttcaatgcttggagatg
ABCC1	gtggaattccggaactac	cggaggtcgtgcaggccg
ABCC2	acagaggctggtggcaacc	accattaccttgtcactgtccatga
ABCG2	tgcaacatgtactggcgaaga	tcttccacaagccccagg
Bmi-1	cagcaatgactgtgatgcact	ccttctccaggtataaatgtaggc
ALDH1A1	agccttgtcctgaagacacc	ttacaaagccgaaacctgtg
β-actin	attgccgacaggatgcaga	gagtacttgcgctcaggagga
