# Supplementary material for: The Clinical Relevance of the miR-197/CKS1B/STAT3-mediated PD-L1 Network in Chemoresistant Non-small-cell Lung Cancer
Source: Mol Ther. 2015 Feb 10;23(4):717–27. doi: 10.1038/mt.2015.10 (PMC4395779; doi:10.1038/mt.2015.10)
Supplement: Supplementary Methods [file mt201510x2.pdf]

Supplementary Methods

Reagents. Mouse monoclonal anti-Cks1 (4G12G7) was purchased from Invitrogen (Carlsbad, CA). Rabbit polyclonal anti-PD-L1 (4059) was purchased from ProSci (Poway CA). Mouse monoclonal anti-STAT3 (124H6), rabbit monoclonal anti-P-STAT3 (Tyr705) (D3A7), mouse monoclonal anti-cyclin D1 (DCS6) were purchased from Cell Signaling Technology (Danvers, MA). Mouse monoclonal anti-Bcl-2 (C-2) (sc-7382) was purchased from Santa Cruz Biotechnology (Dallas, TX). Rabbit monoclonal anti-c-Myc (ab32072) was purchased from Abcam (Cambridge, UK). Mouse monoclonal anti-PD-L1-APC (clone MIH1) and mouse monoclonal IgG1 K isotype control APC antibody (17-4714) was purchased from eBioscience (San Diego, CA). Peroxidase-labeled anti-mouse and anti-rabbit antibodies were included in the Amersham Biosciences ECL PLUS Western blotting reagents pack (RPN2124) (GE HealthCare, UK). Synthetic hsa-miR-197 (pre-miR-197) (5’-CGGGUAGAGAGGGCAGUGGGAGG-3’ and 5’-UUCACCACCUUCUCCACCCAGC-3’) was obtained from the Bonac (Kurume, Japan). The Allstars Negative Control siRNA (1027281) was purchased from Qiagen (Hilden, Germany). miRCURY LNA Power Inhibitor hsa-miR-197 (426907-00) and LNA Power Inhibitor hsa-miR-197-3p-Scrumble Control (199998-00), and LNA Power Inhibitor Negative Control A (199020-00) were purchased from Exiqon (Copenhagen, Denmark). The duplexes of siRNA targeting human STAT3 mRNA was purchased from Cell Signaling Technology (#6580). The siRNAs targeting human CKS1B mRNA (target sequences of 5’-GGAGGAAGCAUCUGAGUUUdTdT3’ and 5’-AAACUCAGAUGCUUCCUCCdTdT3’) was purchased from the Bonac. Cisplatin (CDDP) was obtained from Yakult Honsha Co., Ltd. (Tokyo,

Japan). Paclitaxel (Taxol; TXL) was obtained from Bristol-Myers Squibb (New York, NY). Geneticin and puromycin were purchased from Invitrogen.

Plasmids. pLucNeo was constructed by inserting a firefly luciferase gene derived from the pGL3-control (Promega, Madison, WI) into the pEYFP-1 vector (Clontech Laboratories, Mountain View, CA) at the Bgl*II* and Afl*II* sites. Tough Decoy miRNA-Blocking Expression Vector-miR-197-3p, Tough Decoy miRNA-Blocking Expression Negative Control Vector, and pBApo-CMV pur were purchased from Takara Bio (Shiga, Japan). The sensor vector for miR-197 was constructed by introducing tandem binding sites with perfect complementarity to miR-197 separated by a four-nucleotide spacer into the Xho*I* site of psiCHEAK2 (Promega). The sequences of the binding site are as follows: 5'-AACTCGAGAAGCTGGGTGGAGAAGGTGGTGAAACGCGTAAGCTGGGTGGAGAAGGTGGTGAAAAGCGGCCGCAA-3' (sense) and 5'-TTGCGGCCAGCGGCCGCTTCACCACCTTCTCCACCCAGCAAGAATTCTTTTCACCACCTTCTCCACCCAGCGCGGCCGCTCTAGGTTT-3' (antisense). The "seed" sequence of miR-197 is *underlined*. In a mutated miR-197 sensor vector, the seed sequence, GGTGGTGA, was displaced with CCACCACT. All of the plasmids were verified by DNA sequencing. To generate CTNND1, ERLIN2, CKS1B, CEP55, and PD-L1 luciferase reporter constructs, the 3' UTRs were amplified by PCR and cloned into psiCHECK2 vector at the Xho*I* and Not*I* restriction sites (Promega). Forward primer and reverse primer sequences are as follows (shown 5' to 3'): CTNND1_F, GCTCGAGTAACACTATCTCCGTTCCATCTG and CTNND1_R, GCGGCCGCTAGAGTCTGTAGGTTCTTGATG; ERLIN2_F, GCTCGAGTAAGATGAATCAGAATGTTCCTC and ERLIN2_R, GCGGCCGCTAGCACACCTCTAAAATAACTC; CKS1B_F,

GCTCGAGTAAGCTACTTTTCAGCCTCAAGC and CKS1B_R, GCGGCCGCTAACTTCCTCTTTAATCAAGGC; CEP55_F, GCTCGAGTAAGCTTGTGGGCATTTTGAATT and CEP55_R, GCGGCCGCTAGATTGTAGGTTTTCTAAGTG; PD-L1_F, GCTCGAGTAAAGCGTGACAAGAGGAAGGAA and PD-L1_R, GCGGCCGCTAAGAAAATGGACATGCTGGTG. Site-directed mutagenesis or deletion mutation was performed in the seed sequences of CTNND1, ERLIN2, CKS1B, and CEP55. PrimeStar Max DNA Polymerase (Takara Bio) was used for PCR amplification. Forward primer and reverse primer sequences are as follows (shown 5’ to 3’): CTNND1_Mut_F, TTTGTTTGGGCAATTGACTGATGATTTTC and CTNND1_Mut_R, AATTGCCCAAACAAAAAATAAAAGTACAT; ERLIN2_Mut_F, TACTTTTGGGCAAAAGAAGAAATGAACTT and ERLIN2_Mut_R, TTTTGCCCAAAAGTAACTGGAAGACAGTC; CKS1B_Mut_F, ATGTTTTGGGCCTTGCGGATTTATGTTTC and CKS1B_Mut_R, CAAGGCCCAAAACATACAACACCCGGCAG; CEP55_Mut_F, AGAGTTTGGGCTAGATACTATTTTTTTTT and CEP55_Mut_R, TCTAGCCCAAACTCTTAACAATGGACATC. To examine STAT3 binding to the PD-L1 promoter region, the candidate PD-L1 promoter sequence (-319 bp upstream to +56 bp downstream) was PCR-amplified and ligated into the pGL4.10 [*luc*2] vector at Xho*I* and Bgl*II* restriction sites (Promega). Forward primer and reverse primer sequences are as follows (shown 5’ to 3’): F, CTCGAGCCCGACCCCTCTGAAGGTAAAATC, and R,

AGATCTGCGCGGAAGCTGCGCAGAACTGGG. The sequences of all constructs were confirmed by DNA sequencing.

Establishment of stable cell lines. Stable knockdowns of miR-197 A549 and PC14 cell lines were generated by selection with 4 μg/ml puromycin (Invitrogen). A549 and PC14 cells were transfected with 500 ng of a Tough Decoy miRNA-Blocking Expression Vector-miR-197-3p or a Tough Decoy miRNA-Blocking Expression Negative Control Vector at 90% confluency in 24-well dishes using a Lipofectamine LTX reagent in accordance with the manufacturer's instructions. Stable overexpression of the miR-197 PC14CDDP cell line was generated by selection with 8 μg/ml puromycin. The miR-197 expression plasmid was generated by cloning the genomic pre-miR-197 gene, with a 657-bp sequence on each flanking side, into the BamH*I* and Hind*III* sites of pBApo-CMV pur to generate the plasmid pBApo-miR-197. PC14CDDP cells were transfected with 500 ng of a pBApo-miR-197 or a negative control vector at 90% confluency in 24-well dishes using a Lipofectamine LTX reagent.

Promoter assay. For 3'UTR assay, cells were collected 48 h after cotransfection with each vector and pre-miR-197, and luciferase activity was measured using the Dual-Glo Luciferase Assay System (Promega) according to the manufacturer's protocol. *Renilla* luciferase activity was normalised to firefly luciferase activity. For PD-L1 promoter assay, cells were collected 48 h after cotransfection with the pGL4.10 [*luc*2]-PD-L1 promoter vector, pGL4.74 [*hRluc*/TK] vector, and each siRNA, and luciferase activity was measured using the Dual-Glo Luciferase Assay System (Promega) according to the manufacturer's protocol. Firefly luciferase activity was normalised to *Renilla* luciferase activity.

Culture of lung cancer spheres. The cells were resuspended in 1:1 DMEM/F12 (Invitrogen) basal medium freshly supplemented with 20 ng/mL human basic fibroblast growth factor (Invitrogen), 20 ng/mL epidermal growth factor (Invitrogen), 10 mg/mL heparin (Sigma-Aldrich, St Louis, MO), and 1:50 B27 supplement without vitamin A (Sigma-Aldrich) and seeded in 10-cm Ultra-Low Attachment Surface plates (Corning Inc., Corning, NY) at a density of 1000 cells. To assess the self-renewing potential of the cells, first-generation spheres were collected by gentle centrifugation, dissociated into single-cell suspensions, and cultured under the conditions described above. Ten days later, the plates were analysed for mammosphere formation.

***In situ* hybridisation of miR-197.** The human lung cancer tissues were fixed with Tissue Fixative (Genostaff Co., Ltd. STF-01), embedded in paraffin, and sectioned at 6 μm. For performing *in situ* hybridisation, tissue sections were de-paraffinised with xylene and rehydrated through an ethanol series and PBS. The sections were fixed with 4% para-formaldehyde in PBS for 15 min and then washed with PBS. The sections were then treated with 3 μg/mL of Proteinase K in PBS for 30 min at 37 °C, washed with PBS, re-fixed with 4% para-formaldehyde in PBS, washed again with PBS, and placed in 0.2 N HCl for 10 min. After washing with PBS, the sections were acetylated by incubation in 0.1 M tri-ethanolamine-HCl, pH 8.0, 0.25% acetic anhydride for 10 min. After washing with PBS, hybridisation was performed with Dig labelled LNA probes (Exiqon: has-miR-197-3p or the scramble probe) at concentrations of 18 nM in the Probe Diluent-1 (Genostaff Co., Ltd. RPD-01) at 50 °C for 16 h. After hybridisation, the sections were washed in 2× HybriWash (Genostaff Co., Ltd. SHW-01), equal to 2× SSC, for 15 min and then in 50% formamide, 2× HybriWash at 60 °C for 20 min, three times. The sections were then washed twice with TBST (0.1% Tween 20 in TBS). After treatment with G-block (Genostaff Co., Ltd. GB-01) for 30 min, the sections were incubated with anti–DIG-AP conjugate (Roche, Basel, Switzerland) diluted 1:1,000 with TBST for 2 h at RT.

The sections were washed twice with TBST and then incubated in 100 mM NaCl, 50 mM $MgCl_2$, 0.1% Tween 20, 100 mM Tris-HCl, pH 9.5. Colouring reactions were performed with NBT/BCIP solution (Sigma) overnight and then washed with PBS. The sections were counterstained with Kernechtrot stain solution (Muto pure chemicals, Tokyo, Japan), dehydrated, and then mounted with Malinol (Muto pure chemicals).
